# Aptamer-siRNA chimera and gold nanoparticle modified collagen membrane for the treatment of malignant pleural effusion

**DOI:** 10.3389/fbioe.2022.973892

**Published:** 2022-08-23

**Authors:** Wen Chen, Fengjie Guo, Zhipeng Ren, Linghui Wang, Tinghui Li, Xiaobin Hou

**Affiliations:** ^1^ Department of Pathology, The 8th Medical Center, Chinese PLA General Hospital, Beijing, China; ^2^ Outpatient Department, The 8th Medical Center, Chinese PLA General Hospital, Beijing, China; ^3^ Department of Thoracic Surgery, The First Medical Center, Chinese PLA General Hospital, Beijing, China

**Keywords:** malignant pleural effusion, aptamer, AuNP, PD-1, lung cacer

## Abstract

Malignant pleural effusion is one of the most common complications of advanced lung cancer and there is no effective clinical treatment at present. Here, we constructed an aptamer-siRNA chimeras/PEI/PEG/gold nanoparticle (AuNP)/collagen membrane that can progressively activate T cells by layer by layer assembly. Electron microscope showed this collagen membrane could be divided into 10 layers with a total thickness of 50–80μm, and AuNPs could be observed. Aptamer-siRNA chimeras could bind specifically to OX40^+^ cells and silencing programmed death receptor-1 (PD-1) gene. *In vitro* experiments demonstrated that chimeras/PEI/PEG/AuNPs gradually activated T cells to continuously kill lung adenocarcinoma cells in malignant pleural effusion. Animal experiments showed that chimeras/PEI/PEG/AuNP/collagen membrane effectively treated malignant pleural effusion. Compared with PD-1 inhibitor group, the number of cancer cells, ki-67 proliferation index and CD44 expression in the pleural effusion was significantly decreased and the lymphocyte/cancer cell ratio was significantly increased in the chimeras/AuNP-CM group. Flow cytometry showed that compared with PD-1 inhibitor group, T cell number in the chimeras/AuNP-CM group was significantly increased, while the proportion of PD-1^+^ T cells was markedly decreased. In conclusion, we constructed an chimeras/PEI/PEG/AuNP/collagen membrane, which was more effective in the treatment of malignant pleural effusion, and had less side effects than PD-1 inhibitors.

## Introduction

Lung cancer is one of the malignancies with the highest morbidity and mortality in the world, and many patients have already metastasized when diagnosed. Malignant pleural effusion is defined as the detection of malignant cells or tissues in pleural effusion. Malignant pleural effusion occurs in over 60% of patients with advanced lung cancer, and these patients have a poor prognosis (Dipper and Maskell, 2020). At present, clinical palliative treatment is mainly used, including thoracic drainage and pleural fixation, etc., but the therapeutic effect is not ideal (Shiroshita et al., 2020). Immunotherapy is the most promising treatment for cancer (Wei et al., 2022). Programmed death receptor-1 (PD-1) and programmed death-ligand 1 (PD-L1) are important immune checkpoints. Large-scale clinical studies have demonstrated that PD-1 inhibitors significantly improve overall survival, overall response rate and progression-free survival in patients with advanced non-small cell lung cancer (NSCLC), and their efficacy is superior to chemotherapy (Davis et al., 2021; Mielgo-Rubio et al., 2021).

Malignant pleural effusion is caused by the pleura invasion of malignant tumor, which may cause dyspnea, chest pain and chest tightness. Malignant pleural effusion has been proved to be an immunosuppressive microenvironment. A large number of lymphocytes enter the pleural cavity during the formation of pleural effusion; however, their anti-tumor immune activity is significantly reduced. Activating these lymphocytes *in vitro* can significantly enhance their tumor killing ability and effectively alleviate the occurrence of malignant pleural effusion (He D. et al., 2021). Tumors and inflammation can stimulate pleural thickening and fibrosis, and systemic drugs are difficult to accumulate in the pleural cavity at concentrations that can effectively kill cancer cells. In addition, lymphocytes, nutrients and oxygen in the blood are hard to replenish quickly and consistently. Local application of immunopotentiating agents can rapidly activate lymphocytes in the pleural cavity and rapidly kill cancer cells in a short time. However, rapidly proliferating lymphocytes consume a large number of nutrients and oxygen, forming a local hypoxic and nutriment-deficient microenvironment in the pleural cavity, causing the death of lymphocytes and tissue cells, and may accelerate the progress of lung cancer (Aggarwal et al., 2018; Wahla et al., 2019). Controlled release of immunotherapy drugs, and progressive activation of lymphocytes may be a better method to treat malignant pleural effusion.

**SCHEME 1:**
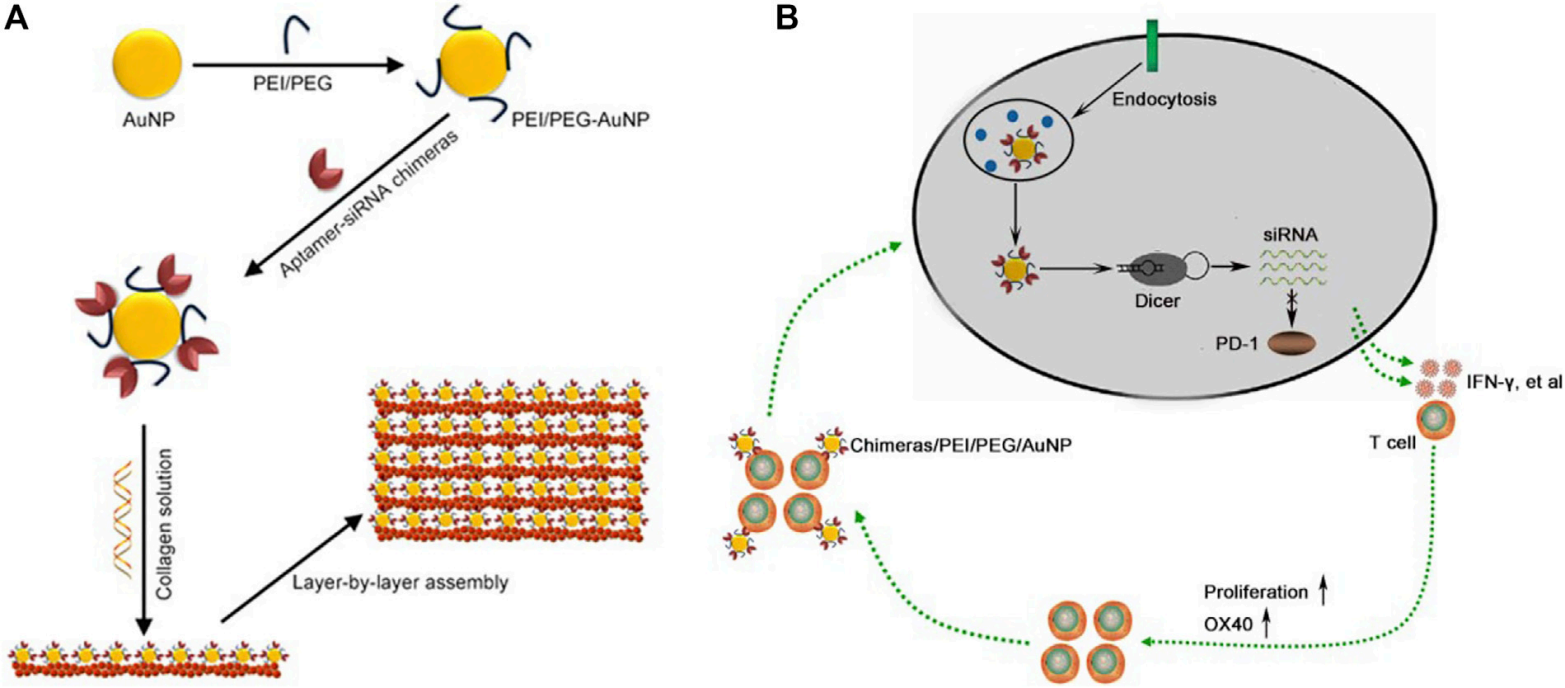
Construction of aptamer-siRNA chimeras/PEI/PEG/AuNP/collagen membrane. **(A)** Schematic diagram. **(B)** Aptamer can specifically bind to activated T cells, and then the bound cells internalized aptamer-siRNA chimeras/PEI/PEG/AuNPs. Chimeras were cut into PD-1 siRNA by Dicer and the RNA-induced silencing complex (RISC) that contained a PD-1 siRNA strand mediated targeted mRNA degradation, further resulting in depletion of PD-1. The slowly released chimeras from collagen membrane continued to bind to the newly activated T cells, thus achieving progressive activation of T cells.

Aptamer is a nucleotide sequence that can bind proteins and cells with high affinity and specificity. Dassie JP et al. designed an aptamer-siRNA chimeras, which can bind and enter the target cell, and form siRNA under the splicing of intracellular Dicer enzyme (Dassie et al., 2009). We have also constructed an aptamer-siRNA chimera, which can specifically capture endothelial progenitor cells and deliver siRNA, with good stability and strong gene silencing effect *in vivo* (Chen et al., 2015). Gold nanoparticles (AuNPs) can be produced via the formation of an Au-amine complex. Recalling the reductive capability of amines, PEI-capped AuNPs would then be able to form. AuNPs have unique advantages in tumor immunotherapy, including immune regulation and inhibition of tumor angiogenesis (He J. S. et al., 2021). In addition, AuNPs can effectively deliver siRNA and immune agents, reduce adverse drug reactions and improve the effectiveness of immunotherapy (Ahn et al., 2014; Perche et al., 2016). In this paper, we constructed an aptamer-siRNA chimeras/PEI/PEG/AuNP/collagen membrane by layer by layer assembly for the treatment of malignant pleural effusion (Scheme 1).

## Materials and methods

### Construction of aptamer-siRNA chimeras/PEI/PEG/AuNP/collagen membrane

Polyethylenimine (PEI, Sigma) was fully dissolved in KH_2_PO_4_/NaOH solution. Then Polyethylene glycol (PEG)-NHS (Sigma) was added and slowly stirred for 2 days 1.44 ml PEI/PEG solution was added into 25 ml HAuCl_4_ aqueous solution (42 mM, Sigma) and slowly stirred for 1 day. Then centrifuged at 12000g for 10 min, discarded the supernatant, and washed with deionized water for three times. OX40 aptamer-PD-1 siRNA chimeras or PD-1 siRNA (synthesized by GenePharma) were added, stirred gently in dark for half an hour, centrifuged at 12000g for 10 min, and discarded the supernatant. After washing with deionized water for three times, aptamer-siRNA chimeras/PEI/PEG/AuNP nanoparticles were obtained.

10 mg aptamer-siRNA chimeras/PEI/PEG/AuNP nanoparticles were dissolved in 10 ml collagen solution (Sigma) and stirred slowly at 4°C for 2 h. Then 150 uL collagen solution was carefully drizzled onto the glass slide and then freeze-dried to form the monolayer membrane. Chimeras/PEI/PEG/AuNP/collagen monolayer membrane was put into heparin solution (1 mg/ml, Sigma). After 15 min, another monolayer membrane was gently put on the top of first monolayer membrane and cross-linked for 15 min. After 10 repetitions, collagen membranes were carefully washed with ionized water for three times. In the end, freeze drying was performed to obtain chimeras/PEI/PEG/AuNP/collagen membranes.

### Release experiment

Carboxyfluorescein (FAM)-chimeras/PEI/PEG/AuNP/collagen membrane was placed into PBS and gently shaken in the dark. The collagen membrane was removed and placed into activated T cells at day 1, 2, 4, 7, 9, 11, and 14. After 12 h, T cells in each group were collected, added with OX40-phycoerythrin (PE), and incubated at room temperature and in dark for 15 min. After washing with PBS for three times, the proportion of positive cells was detected by flow cytometry. The binding rate was used to calculate the ability of the collagen membrane to release chimeras. Binding rate = FAM^+^PE^+^ cells/PE^+^ cells.

### Flow cytometry

Malignant pleural effusions from patients with advanced lung adenocarcinoma were collected and divided into two groups on average: PD-1 inhibitor (PD-1 inhibitor nivolumab was added) and chimeras/AuNP group (OX40 aptamer-PD-1 siRNA chimeras/PEI/PEG/AuNPs was added). The pleural effusion of each group was collected before treatment, 1 day, 2, 3 and 4 days later, and centrifuged at 1500 g for 5 min. The supernatant was discarded, CD3-PE (BD) was added, and incubated in dark for 15 min. After washing with PBS for three times, the proportion of CD3^+^ T cells was determined by flow cytometry.

### ELISA detection

Malignant pleural effusions from patients with advanced lung adenocarcinoma were collected and divided into two groups on average: PD-1 inhibitor (PD-1 inhibitor nivolumab was added) and chimeras/AuNP group (OX40 aptamer-PD-1 siRNA chimeras/PEI/PEG/AuNPs was added). The pleural effusion of each group was collected before treatment, 1 day, 2, 3 and 4 days later, and centrifuged at 1500 g for 5 min. The supernatant was discarded and the cells were cultured in RPMI1640 containing 10% fetal bovine serum (Hyclone). After 24 h, the supernatant was collected and the concentration of IFN-γ was determined by ELISA (Xiamen Huijia). The experiment was carried out in strict accordance with the kit instructions.

### Apoptosis experiment

The lung adenocarcinoma cell line A549 (ATCC) was cultured in RPMI1640 containing 10% fetal bovine serum. A549 at logarithmic growth stage was digested with 0.25% trypsin (Hyclone) and divided into five parts on average. Malignant pleural effusion of patients with advanced lung adenocarcinoma was collected and divided into five groups on average: Control group (adding PBS), PD-1 inhibitor group (adding PD-1 inhibitor nivolumab), AuNP group (adding AuNPs), siRNA/AuNP group (adding PD-1 siRNA/PEI/PEG/AuNPs), Chimeras/AuNP group (adding OX40 aptamer-PD-1 siRNA chimeras/PEI/PEG/AuNPs). After 48 h or 96 h, the supernatant was discarded after centrifugation at 1500 g for 5 min. After washing with PBS for three times, cells in each group were collected and added into the medium of A549. After 48 h of co-culture, the supernatant was discarded, washed three times with PBS, and A549 cells in each group were collected. AnnexinV-fluorescein-5-isothiocyanate (FITC) and propidium iodide (PI, Roche) were added, and the cells were incubated in dark for 15 min, then 400 μL binding buffer (Roche) was added, and the proportion of apoptotic cells was detected by flow cytometry.

### MTT proliferation assay

After 48 h of cell co-culture, the supernatant was discarded and washed three times with PBS. A549 cells in each group were collected and planted in 96-well plates at a density of 4x10^3^ per well. 24 h later, 5 mg/ml MTT (Sigma) was added and the culture was continued for 4 h. The supernatant was discarded and added 150 µL DMSO (Sigma). After low-speed oscillation for 10 min, the absorbance value (OD: 490 nm) of each well was measured at the enzyme-linked immunoassay, and the proliferation rate of each group was calculated.

### Animal experiment

6-week-old C57BL/6J wild-type mice were anesthetized with 1% sodium pentobarbital. A549 cells (2×10^5^ cells suspended in 50 μL of PBS) were injected slowly into the right thoracic cavity at midaxillary level. Four days after the injection of A549 cells, mice were randomly divided into five groups: Control group (injection of PBS into the right pleural cavity), PD-1 inhibitor group (injection of 1 mg/kg PD-1 inhibitor into the right pleural cavity), AuNP-CM group (implantation of AuNP/collagen membrane in the right pleural cavity), siRNA/AuNP-CM group (implantation of PD-1 siRNA/PEI/PEG/AuNP/collagen membrane in the right pleural cavity) and chimeras/AuNP-CM group (implantation of OX40 aptamer-PD-1 siRNA chimeras/PEI/PEG/AuNP/collagen membrane in the right pleural cavity). After 2 weeks, the mice were anesthetized with 1% sodium pentobarbital. Pleural effusion was collected in each group, and rinsed repeatedly and the rinsing fluid was collected. Cell smears were made to observe the number of lymphocytes and cancer cells. After centrifugation at 1500 g for 10 min, cell blocks were prepared, and then Hematoxylin and Eosin (H&E) staining was performed. The expression of CD44 and Ki-67 (Abcam) were detected by immunohistochemistry. All animal experiments were performed in accordance with the regulations on animal experiments of Chinese PLA General Hospital (Beijing, China) and were approved by the Ethics Committee of Chinese PLA General Hospital (2020-08).

### Statistical methods

Statistical analyses were performed using Graphpad Software 5.0. Multiple data comparisons were performed via One-Way ANOVA and Bonferroni post hoc test. Comparison between the two groups was performed using Student’s t test. *p* < 0.05 was considered statistically significant.

## Results

### The characteristics of chimeras/PEI/PEG/AuNP/collagen membranes

**FIGURE 1:**
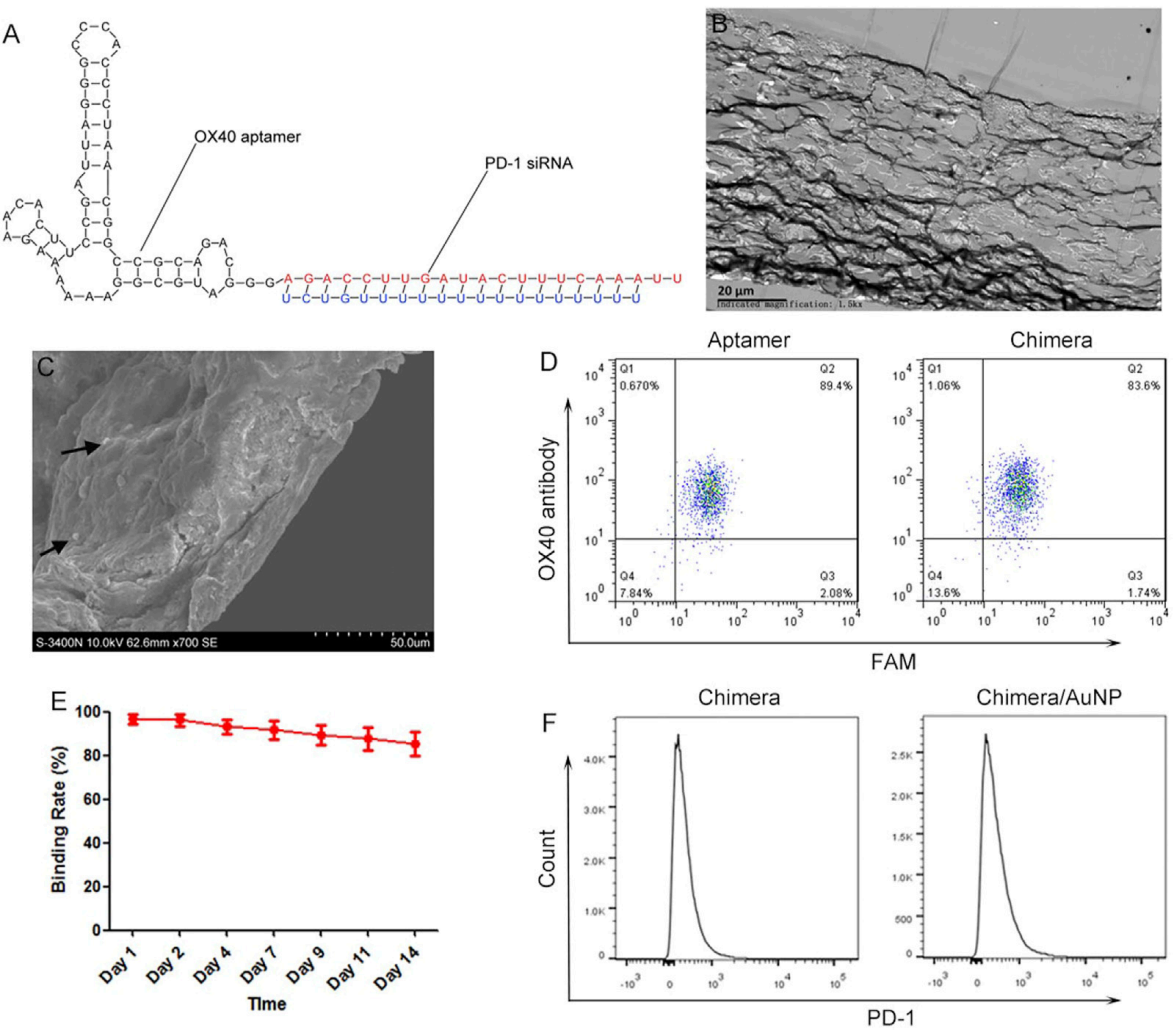
Characteristics of aptamer-siRNA chimeras/PEI/PEG/AuNP/collagen membrane **(A)** Predicted secondary structure for OX40 aptamer-PD-1 siRNA chimera **(B)** Transmission electron microscope images **(C)** Scanning electron microscope images **(D)** We used OX40 antibody as the positive control, and flow cytometry results showed that both OX40 aptamer and OX40 aptamer-PD-1 siRNA chimera could bind to OX40^+^ cells with high specificity **(E)** Release experiments showed that chimeras/PEI/PEG/AuNP/collagen membrane could control the release of chimeras for at least 2 weeks **(F)** Both OX40 aptamer-PD-1 siRNA chimera and chimera/AuNP significantly inhibited the expression of PD-1. Chimeras/PEI/PEG/AuNPs progressively activated T cells in malignant pleural effusion.

OX40 is an important marker of T cell activation, and its aptamer can promote the proliferation of T cells and maintain the survival of T cells. The constructed chimera can be divided into two parts (Figure 1A). OX40 aptamer can bind specifically to activated T cells and deliver siRNA. PD-1 siRNA can silence PD-1 gene, activate T cells, and further activate surrounding T cells by releasing inflammatory cytokines, forming a virtuous cycle of gradual activation of T cells. Transmission electron microscope showed that chimeras/PEI/PEG/AuNP/collagen membrane could be divided into 10 layers with a total thickness of 50–80 μm in cross section (Figure 1B). Scanning electron microscope results also showed that the thickness of chimeras/PEI/PEG/AuNP/collagen membrane was 50–80μm, and AuNPs could be observed. The size of aptamer-siRNA chimeras/PEI/PEG/AuNPs was 150–300 nm and their zeta-potential was about 10.3 mV (Figure 1C). The loading ratio of aptamer-siRNA chimeras in the AuNPs was 36.57%. We used OX40 antibody as the positive control, and flow cytometry results showed that both OX40 aptamer and OX40 aptamer-PD-1 siRNA chimera could bind to OX40^+^ cells with high specificity (Figure 1D). After 14 days, chimeras binding to OX40^+^ cells were still detected in PBS, indicating that chimeras/PEI/PEG/AuNP/collagen membrane could control the release of chimeras for at least 2 weeks (Figure 1E). Flow cytometry results showed that both OX40 aptamer-PD-1 siRNA chimera and chimera/AuNP significantly inhibited the expression of PD-1 (Figure 1F).

**FIGURE 2:**
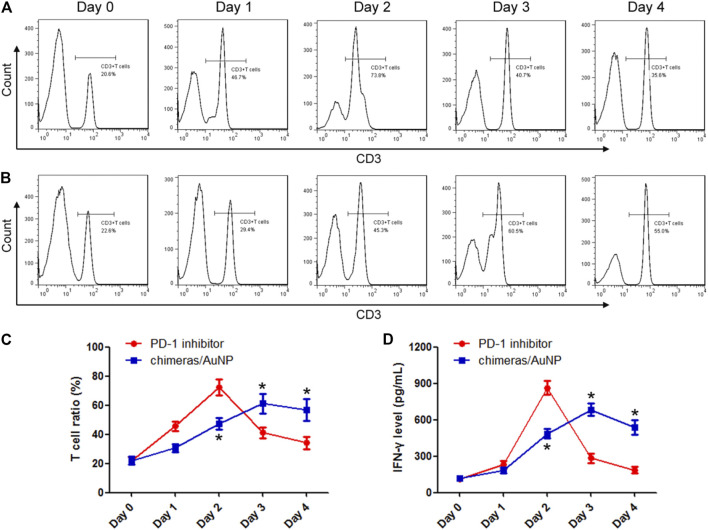
Chimeras/PEI/PEG/AuNPs progressively activated T cells in malignant pleural effusion. Malignant pleural effusions from patients with advanced lung adenocarcinoma were collected and treated with PD-1 inhibitor **(A)** or chimeras/PEI/PEG/AuNPs **(B)**, respectively **(C,D)** PD-1 inhibitor could rapidly activate T cells, T cell proportion and IFN-γ concentration increased rapidly, and reached the peak 48 h later. Then T cell proportion and the concentration of IFN-γ released began to decrease. Chimeras/PEI/PEG/AuNPs slowly activated T cells, and T cell proportion and IFN-γ concentration increased gradually. *p < 0.05 (n = 10) versus PD-1 inhibitor. Values are mean ± SD. Chimeras/PEI/PEG/AuNPs continuously killed lung adenocarcinoma cells.

Malignant pleural effusions from patients with advanced lung adenocarcinoma were collected and treated with PD-1 inhibitor or chimeras/PEI/PEG/AuNPs, respectively. The results showed that the PD-1 inhibitor could rapidly activate T cells, the proportion of T cells and the concentration of IFN-γ increased rapidly, and reached the peak 48 h later. Then the proportion of T cells and the concentration of IFN-γ released began to decrease (Figures 2A,D). Chimeras/PEI/PEG/AuNPs slowly activated T cells, and the proportion of T cells increased gradually. After 48 h of treatment, the proportion of T cells and the concentration of IFN-γ in the chimeras/AuNP group were lower than those in the PD-1 inhibitor group. After 72 and 96 h treatment, the proportion of T cells and the concentration of IFN-γ in the chimeras/AuNP group were significantly higher than those in the PD-1 inhibitor group (Figures 2B,D). These results suggested that compared with PD-1 inhibitors, the effect of chimeras/PEI/PEG/AuNPs was more persistent, milder and may have fewer side effects.

**FIGURE 3:**
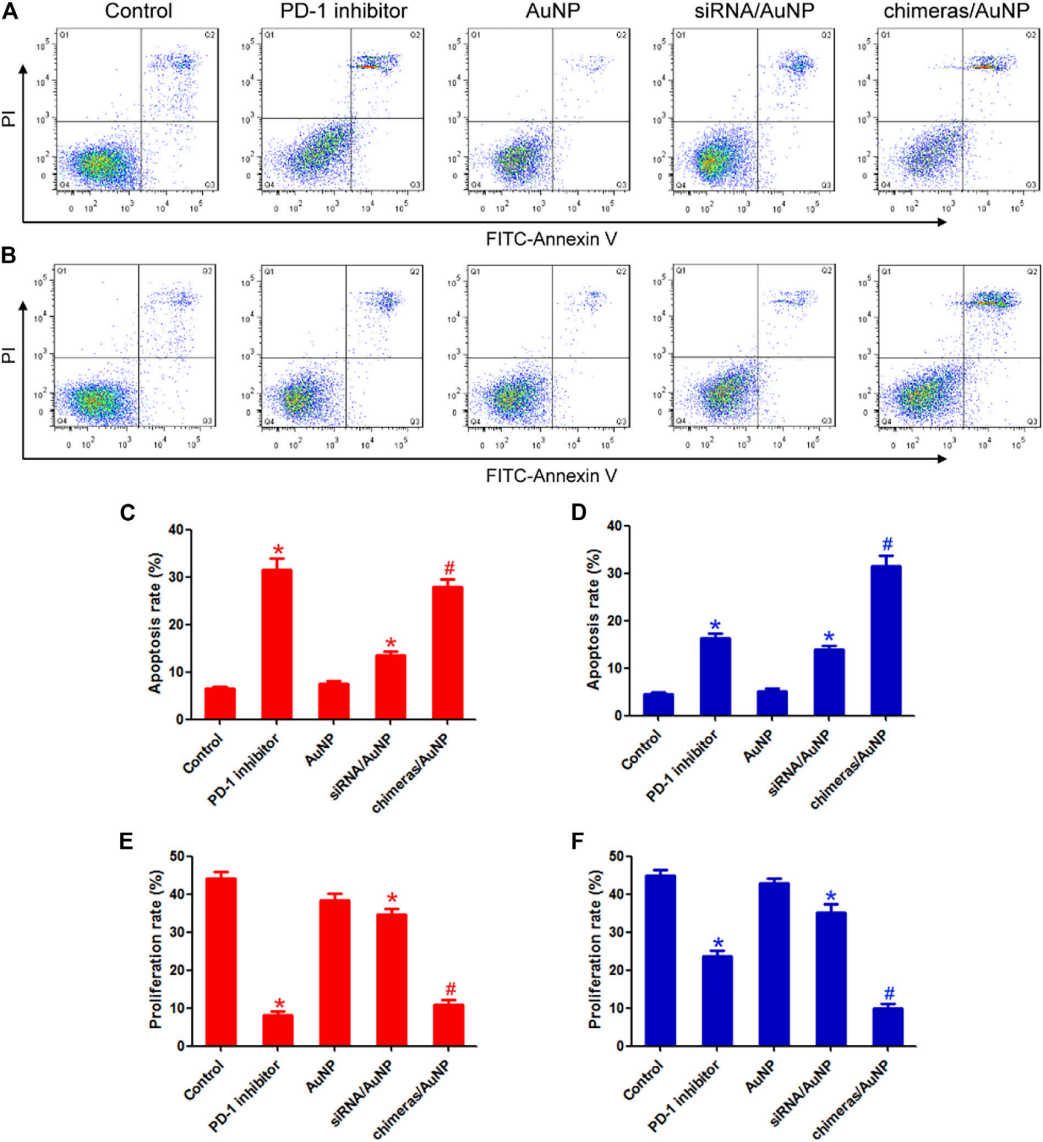
Chimeras/PEI/PEG/AuNPs continuously killed lung adenocarcinoma cells. After treatment for 48 h **(A)** or 96 h **(B)**, lymphocytes were collected and co-cultured with lung adenocarcinoma cells. Flow cytometry was used to detect cell apoptosis **(C,E)** After treatment for 48 h, there were no statistically significant differences in apoptosis rate and proliferation rate between the PD-1 inhibitor group and the chimeras/AuNP group **(D,F)** Compared with PD-1 inhibitor group, the apoptosis rate of lung adenocarcinoma cells in the chimeras/AuNP group was increased by 93.51% and the proliferation level was decreased by 58.46%. *p < 0.05 (n = 10) versus Control. ^#^ *p* < 0.05 (n = 10) versus siRNA/AuNP. Values are mean ± SD. Chimeras/PEI/PEG/AuNP/collagen membrane effectively treated malignant pleural effusion *in vivo*.

After treatment with PD-1 inhibitor or chimeras/PEI/PEG/AuNPs for 48 h, lymphocytes were collected and co-cultured with lung adenocarcinoma cells. There were no statistically significant differences in apoptosis rate and proliferation rate between the PD-1 inhibitor group and the chimeras/AuNP group. Compared with siRNA/AuNP group, the apoptosis rate of lung adenocarcinoma cells in the chimeras/AuNP group was increased by 1.05 times and the proliferation level was decreased by 68.27%. Figures 3A,C,E.

After treatment with PD-1 inhibitor and chimeras/PEI/PEG/AuNPs for 96 h, lymphocytes were collected and co-cultured with lung adenocarcinoma cells. Compared with PD-1 inhibitor group, the apoptosis rate of lung adenocarcinoma cells in the chimeras/AuNP group was increased by 93.51% and the proliferation level was decreased by 58.46%. Compared with siRNA/AuNP group, the apoptosis rate of lung adenocarcinoma cells in the chimeras/AuNP group was increased by 1.26 times and the proliferation level was decreased by 72.04%. These results suggested that compared with PD-1 inhibitors, chimeras/PEI/PEG/AuNPs had better immunotherapeutic effect and longer anti-cancer effect. Figures 3B,D,F.

**FIGURE 4:**
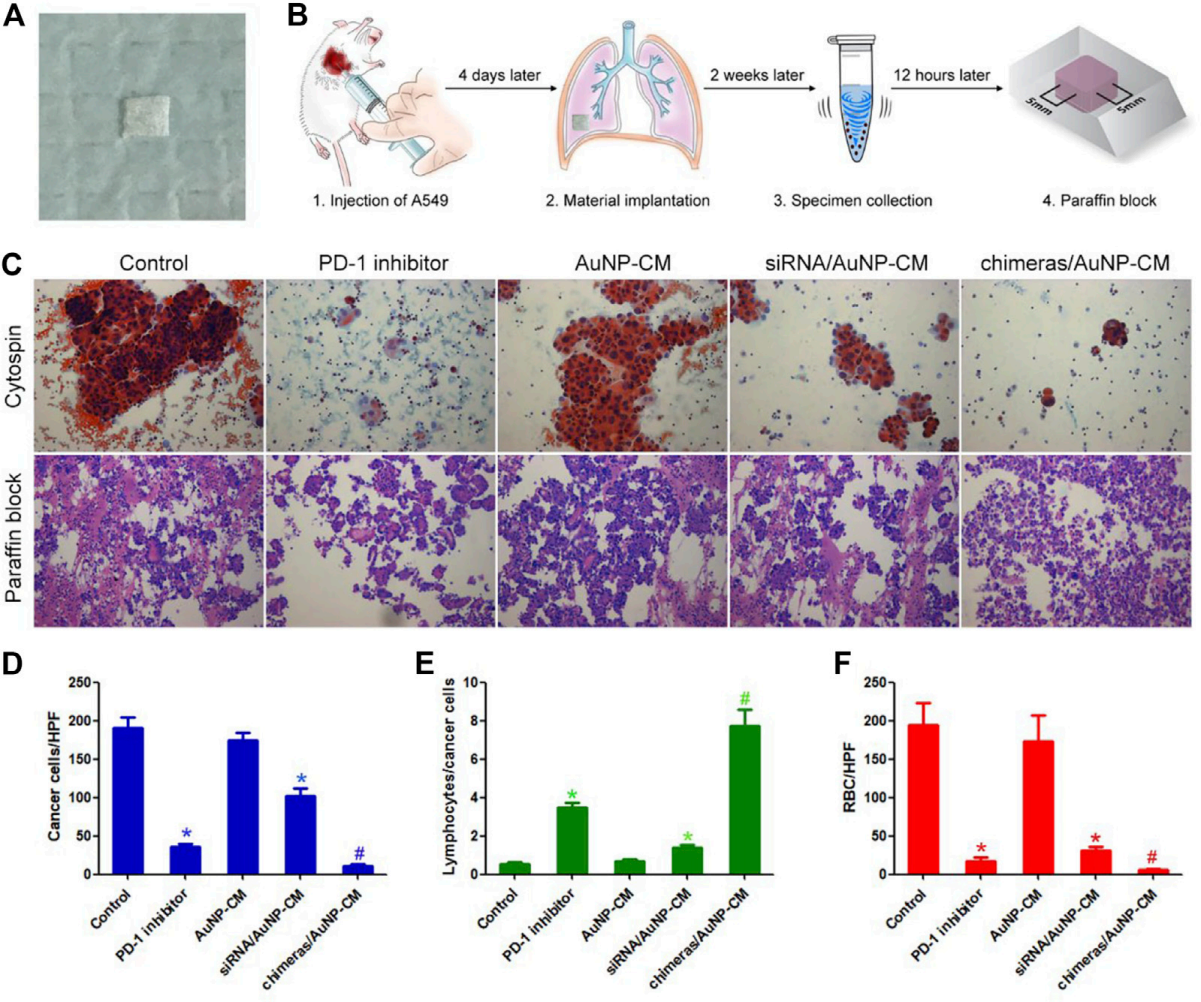
Chimeras/PEI/PEG/AuNP/collagen membrane effectively treated malignant pleural effusion *in vivo* **(A)** General picture **(B)** Flow chart of animal experiments **(C)** Cytospin and HE staining of cell blocks **(D)** Compared with the PD-1 inhibitor group, the number of cancer cells per high power field (HPF) in pleural effusion in the chimeras/AuNP-CM group were reduced by 71.31% **(E)** Compared with the PD-1 inhibitor group, lymphocyte/cancer cell ratio in pleural effusion in the chimeras/AuNP group was increased by 1.20 times **(F)** Compared with the siRNA/AuNP-CM group, the number of red blood cells per high power field (RBC/HPF) was markedly reduced in the chimeras/AuNP-CM group. *p < 0.05 (n = 10) versus Control. ^#^ *p* < 0.05 (n = 10) versus siRNA/AuNP-CM. Values are mean ± SD.

Animal model of malignant pleural effusion was established to verify the effect of chimeras/PEI/PEG/AuNP/collagen membrane *in vivo*. Cell smear results showed that the number of cancer cells in pleural effusion in the PD-1 inhibitor group were reduced by 81.08%, compared with the control group. Compared with the PD-1 inhibitor group, the number of cancer cells in pleural effusion in the chimeras/AuNP-CM group were reduced by 71.31%. We further calculated the lymphocyte/cancer cell ratio. Lymphocyte/cancer cell ratio in pleural effusion in the PD-1 inhibitor group was increased by 5.65 times, compared with the control group. Compared with the PD-1 inhibitor group, lymphocyte/cancer cell ratio in pleural effusion in the chimeras/AuNP group was increased by 1.20 times. In addition, a large number of red blood cells were observed in the control and AuNP-CM groups, indicating that the cancer cells had damaged blood vessels. Some red blood cells and necrotic matter were observed in the PD-1 inhibitor group, while almost none were observed in the chimeras/AuNP-CM group. Figure 4.

**FIGURE 5:**
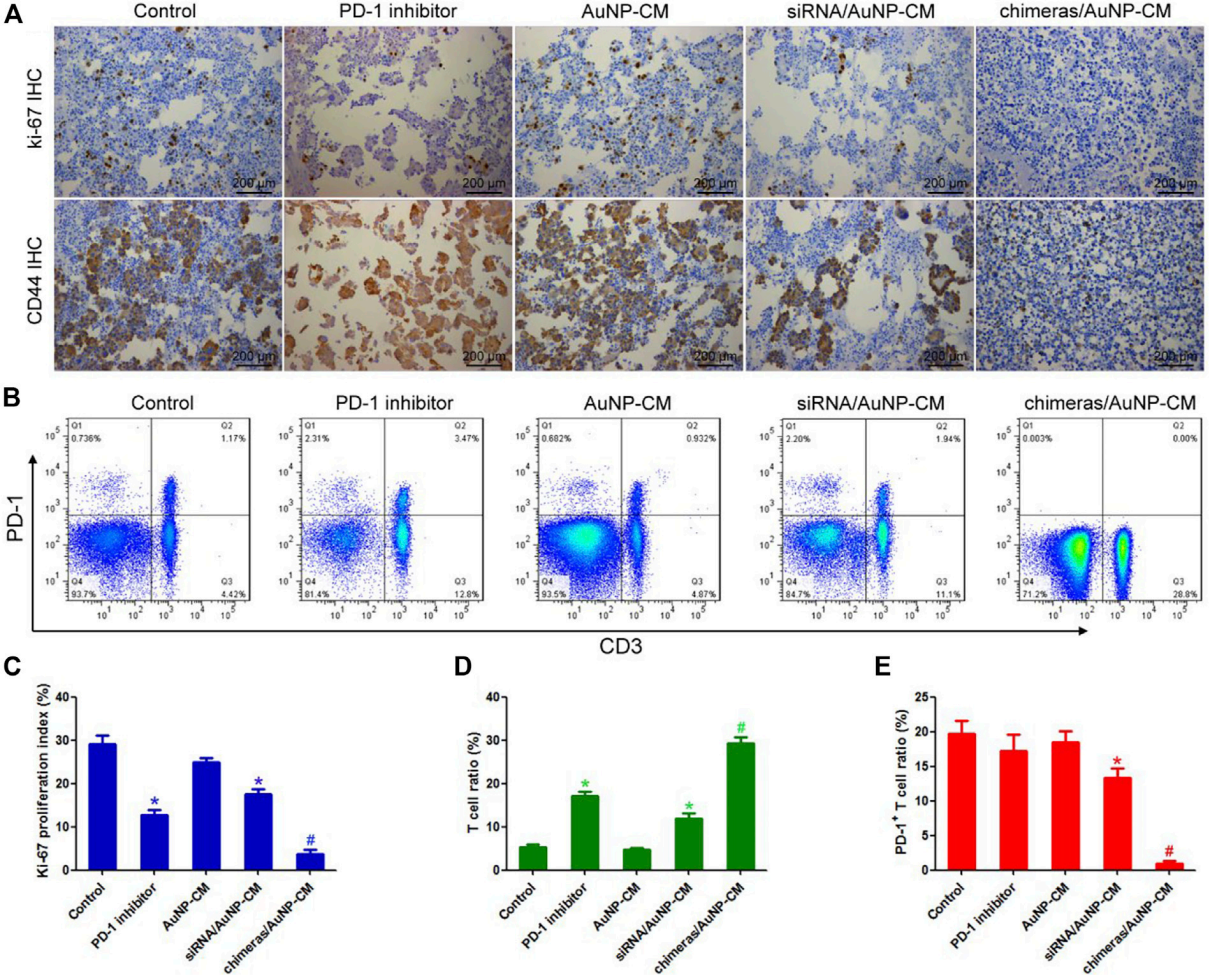
Effects of chimeras/PEI/PEG/AuNP/collagen membrane on immunotherapy for malignant pleural effusion **(A)** Immunohistochemistry was used to detect the expression of Ki-67 and CD44 **(B)** The proportion of T cells and PD-1^+^T cells in pleural effusion was detected by flow cytometry **(C)** Compared with PD-1 inhibitor group, ki-67 proliferation index and CD44 expression of cancer cells in the chimeras/AuNP-CM group were significantly decreased **(D,E)** Compared with the PD-1 inhibitor group, the number of T cells in the chimeras/AuNP-CM group was significantly increased, while the proportion of PD-1^+^T cells was significantly decreased. *p < 0.05 (n = 10) versus Control. ^#^ *p* < 0.05 (n = 10) versus siRNA/AuNP-CM. Values are mean ± SD.

Ki-67 is the main marker of cell proliferation in clinicopathology (Al-Keilani et al., 2021), while CD44 is an important marker of tumor invasion and metastasis (Shinohara et al., 2016). Immunohistochemical results showed that ki-67 proliferation index and CD44 expression were significantly decreased in PD-1 inhibitor group, compared with the control group. Compared with PD-1 inhibitor group, ki-67 proliferation index and CD44 expression of cancer cells in the chimeras/AuNP-CM group were further significantly decreased (Figures 5A,C). Finally, the proportion of T cells and PD-1^+^T cells in pleural effusion was detected by flow cytometry. The results showed that compared with the control group, the proportion of T cells in the PD-1 inhibitor group was significantly increased, but there was no significant difference in the proportion of PD-1^+^T cells. Compared with the PD-1 inhibitor group, the number of T cells in the chimeras/AuNP-CM group was significantly increased, while the proportion of PD-1^+^T cells was markedly decreased (Figures 5B,D,E). These results indicated that chimeras/PEI/PEG/AuNP/collagen membrane was more effective in the treatment of malignant pleural effusion, and had less side effects than PD-1 inhibitors.

## Discussion

In recent years, the global incidence of lung cancer is increasing year by year, and NSCLC is the most common pathological type. Surgical treatment is the most effective treatment for early NSCLC. Advanced lung cancer is often treated with comprehensive therapy, and immunotherapy is considered to be the most promising treatment (Steuer and Ramalingam, 2021). However, only <15% of patients could acquire an objective benefit from immunotherapy, and immune-related adverse events are frequent and sometimes severe. Li et al. recommended that nanomedicine should be desired to improve not only the safety but also the efficacy of immunotherapy (Li and Kataoka, 2021). For malignant pleural effusion, tumors and inflammation that stimulate pleural thickening and fibrosis, PD-1 inhibitors are difficult to enter the pleural cavity from peripheral blood and accumulate in sufficient drug concentrations (He D. et al., 2021). Our results showed that intrapleural administration of PD-1 inhibitors rapidly activated T cells and had a high anti-tumor effect in the short term. After malignant pleural effusion, the pleural cavity is a relatively nutrient and oxygen-deficient microenvironment. The rapidly proliferating lymphocytes consume a large amount of oxygen and nutrients, and gradually begin to die and the anti-tumor activity decreases after 2 days. To solve this clinical problem, we constructed a collagen membrane that can slowly activate T cells, and have a longer lasting effect and better anti-tumor effect than PD-1 inhibitors.

Compared with the antibody, the aptamer has the advantages of good targeting, strong modification, convenient synthesis and low price. Several aptamers have been approved by the FDA to treat diseases such as age-related macular degeneration (Siddiqui and Keating, 2005). Aptamers can realize the aggregation of drugs at the lesion site without affecting the normal tissue, thus reducing the toxic and side effects of drugs (Tang et al., 2020). In this paper, we combine aptamer and siRNA to construct a nucleic acid chimera. OX40 is an important marker of T cell activation (Ohmura et al., 2020; Yan et al., 2021), and our experimental results showed that both OX40 aptamer and chimera could efficiently bind to OX40^+^ cells. After combining with activated T cells, the OX40 aptamer can, on the one hand, promote the proliferation of T cells and maintain their survival in the nutrient-deficient and hypoxic microenvironment (Pratico et al., 2013), on the other hand, deliver PD-1 siRNA into T cells to silence PD-1 gene. Blockage of PD-1 can effectively activate T cells and release cytokines to further activate surrounding T cells. The slowly released chimeras continued to bind to the newly activated T cells, thus achieving progressive activation of T cells. Side effects of immunotherapy are mainly caused by tumor necrosis and activated immune cells, which may cause inflammatory damage to other normal organs. Our results showed that compared with PD-1 inhibitors, the effect of chimeras/PEI/PEG/AuNPs was more persistent and milder, so there’s less damage to normal organs. Our study provides a new method for immunotherapy of malignant tumors in a relatively closed microenvironment.

Here, we constructed an aptamer-siRNA chimeras/PEI/PEG/AuNP/collagen membrane by layer by layer assembly for the treatment of malignant pleural effusion. Our collagen membrane had the following advantages: 1) OX40 aptamer-PD-1 siRNA chimeras can progressively activate T cells while protecting them. These chimeras exhibited better and more durable antitumor activity than PD-1 inhibitors in the microenvironment of relative nutrient deficiency and hypoxia. 2) The novel drug administration method. Our collagen membrane can control the continuous release of nucleic acid chimeras for more than 14 days, reducing the trauma and other side effects caused by repeated drug administration. Clinicians can implant collagen membrane through endoscopic surgery to prevent and treat malignant pleural effusion. Many factors may affect the release of the aptamer-siRNA chimeras/PEI/PEG/AuNP from the collagen membrane, including the rate of collagen degradation, the level of inflammation, macrophages, and some enzymes or ions in body fluids. Collagen degradation and inflammation may be the main mechanisms. 3) AuNPs can enhance the effectiveness of immunotherapy and reduce side effects. PEG modification could help to make stable and uniform structure of nanoparticles (Li and Kataoka, 2021). 4) We can replace this drug delivery system with the corresponding aptamer and siRNA according to the tumor type and immune checkpoint, so as to achieve the purpose of precision therapy.

## Conclusion

In conclusion, we constructed an OX40 aptamer-PD-1 siRNA chimeras/PEI/PEG/AuNP/collagen membrane that can effectively treat malignant pleural effusion. Animal experiments showed that this collagen membrane had fewer side effects than immune checkpoint inhibitors. Our study provides a new method for the immunotherapy of malignant tumors in a relatively closed microenvironment.

## Data Availability

The original contributions presented in the study are included in the article/Supplementary Material, further inquiries can be directed to the corresponding authors.
